# Evaluating Health Workers' Potential Resistance to New Interventions: A Role for Discrete Choice Experiments

**DOI:** 10.1371/journal.pone.0023588

**Published:** 2011-08-19

**Authors:** Mylene Lagarde, Lucy Smith Paintain, Gifti Antwi, Caroline Jones, Brian Greenwood, Daniel Chandramohan, Harry Tagbor, Jayne Webster

**Affiliations:** 1 Department of Global Health and Development, London School of Hygiene and Tropical Medicine, London, United Kingdom; 2 Department of Disease Control, London School of Hygiene and Tropical Medicine, London, United Kingdom; 3 School of Medical Sciences, Kwame Nkrumah University of Science and Technology (KNUST), Kumasi, Ghana; Mahidol University, Thailand

## Abstract

**Background:**

The currently recommended approach for preventing malaria in pregnancy (MiP), intermittent preventive treatment with sulphadoxine-pyrimethamine (SP-IPT), has been questioned due to the spread of resistance to SP. Whilst trials are underway to test the efficacy of future alternative approaches, it is important to start exploring the feasibility of their implementation.

**Methods and Findings:**

This study uses a discrete choice experiment (DCE) method to assess the potential resistance of health workers to changing strategies for control of MiP. In Ashanti region in Ghana, 133 antenatal clinic health workers were presented with 16 choice sets of two alternative policy options, each consisting of a bundle of six attributes representing certain clinical guidelines for controlling MiP (type of approach and drug used), possible associated maternal and neo-natal outcomes, workload and financial incentives. The data were analysed using a random effects logit model. Overall, staff showed a preference for a curative approach with pregnant women tested for malaria parasites and treated only if positive, compared to a preventive approach (OR 1.6; p = 0.001). Increasing the incidence of low birth weight or severe anaemia by 1% would reduce the odds of preferring an approach by 18% and 10% respectively. Midwives were more resistant to potential changes to current guidelines than lower-level cadres.

**Conclusions:**

In Ashanti Region, resistance to change by antenatal clinic workers from a policy of SP-IPT to IST would generally be low, and it would disappear amongst midwives if health outcomes for the mother and baby were improved by the new strategy. DCEs are a promising approach to identifying factors that will increase the likelihood of effective implementation of new interventions immediately after their efficacy has been proven.

## Introduction

Efficacy studies are critical to answer the question of whether a new intervention is superior or non-inferior to current policy guidelines in achieving the desired health outcomes under trial conditions. These studies may be implemented for interventions that are not yet policy within a given country, for example when there are signs suggesting that a current intervention is failing and a replacement is needed. They generally take place on a small scale, in defined geographic areas, and under carefully controlled conditions, with stringent data checking procedures to ensure the safety of participants. Whilst they may provide internally valid evidence on the efficacy of an intervention, efficacy studies do not evaluate the feasibility of implementation of an intervention under operational conditions.

There is a growing body of literature on the failure of delivery, and missed opportunities for patients to access interventions even when they attend health facilities [1]. Indeed, the translation of efficacy of an intervention under trial conditions to effectiveness under operational conditions is dependent upon the effectiveness of all of the composite processes involved in delivering the intervention. These processes include end-user access to the delivery point of the intervention, provider compliance in delivering the intervention, end-user adherence, and individual benefit from the intervention [2]. Investigation of the reasons why patients are not offered an intervention is essential to identify appropriate interventions to improve the effectiveness of the delivery process.

RCTs take place under controlled conditions and, therefore, cannot be used to forecast the effectiveness of the same processes under routine conditions. Often several of the delivery processes, particularly those involving diagnosis and prescription of drugs, are conducted by trial staff rather than those with responsibility under operational conditions. Provider compliance to guidelines in delivering an intervention is a critical step in ensuring the effectiveness of an intervention.

By their nature, studies investigating the operational feasibility of new interventions require implementation at scale, under ‘real life’ conditions, not in controlled experimental settings. Thus they require that an intervention has been, or may be, rolled out in the health system, and therefore they cannot be conducted for interventions that are not yet within the policy guidelines of a country. This is particularly challenging where a new intervention is proposed as an alternative to an existing intervention; in this case, an operational feasibility study to compare the two approaches would require that those being given the new intervention are deprived of the intervention which is the current national guideline. Therefore, until there is sufficient evidence demonstrating the efficacy of a new treatment, and until this has translated into the modification of national guidelines, research exploring the operational feasibility of the new intervention has to wait.

Translation of efficacious interventions identified in trials into policy is a complex process which takes years rather than months. However, once the policy change is effected there is often pressure to implement quickly rather than await the findings of implementation research. The result is either delayed implementation of new efficacious interventions as barriers to implementation are investigated, or more often, implementation without operational research resulting in an efficacious intervention with significantly lower effectiveness.

Discrete choice experiments (DCEs), a stated preference method, can be used to overcome these challenges by investigating potential barriers to implementation of a new intervention before it has been incorporated into national policy guidelines. They can be conducted whilst RCTs are still underway, since they rely on the examination by respondents of hypothetical scenarios (not on the actual delivery of one intervention over another). DCEs have been widely used by health economists to elicit patients' preferences for different types of health services [3], [4] or to assess providers' preferences for treatment or screening options for patients in high-income settings [5]. Since they allow the quantification of the preferences of health providers for alternative interventions and for specific components of each of these interventions, a promising application of DCEs is to assess potential resistance among heath workers to introduction of new interventions that are likely to be incorporated into national policy guidelines in the near future. The findings may be used to guide operational research studies and to direct the design of delivery strategies to maximise acceptance by the health workers responsible for delivery of the intervention.

*Plasmodium falciparum* infection in pregnancy is associated with an increased risk of maternal and foetal complications including maternal anaemia and low birth weight [6], [7]. Together with insecticide treated nets and effective case management, intermittent preventive treatment during pregnancy with sulphadoxine-pyrimethamine (SP-IPT) is currently recommended by the World Health Organisation [8], and it is in the national policy guidelines for prevention of MiP in Ghana [9] and other countries of West Africa [10]. Yet, the effectiveness of SP-IPT has been questioned due to the spread of SP resistance across sub-Saharan Africa [11]. There is evidence for reduced effectiveness of SP-IPT from East and Southern Africa [12], and studies are underway in West Africa through the MiP Consortium [13]. Doubts regarding the efficacy of current national and international guidelines have recently led to the implementation of trials investigating the efficacy of alternative drugs to SP for IPT and to alternative interventions [12], [13].

An RCT was conducted in the Ashanti Region of Ghana between February 2007 and November 2008 to investigate the efficacy of an alternative intervention to SP-IPT, namely intermittent screening and treatment (IST). In two arms of the trial, pregnant women were screened at scheduled antenatal care (ANC) visits with a rapid diagnostic test (RDT) and those with a positive result were treated with either artesunate-amodiaquine (AS-AQ) or SP; women in the third arm were not screened and received three doses of SP-IPT at monthly intervals as per current national policy. The alternative intervention, IST was found to be non-inferior to the current strategy (SP-IPT) in improving maternal anaemia and birth weight outcomes [14].

In this study, we used a DCE to determine the elements of the current strategy (SP-IPT) and prospective new strategy (IST) for controlling malaria in pregnancy that were important to ANC staff, in order to identify potential obstacles to the implementation of a new strategy and elements that could be used as levers to facilitate change.

## Methods

### Ethics

Ethical approval for this study was granted by the Committee on Human Research, Publications and Ethics, Kwame Nkrumah University of Science and Technology, School of Medical Sciences, Kumasi, Ghana and by the ethics committee of the London School of Hygiene & Tropical Medicine. Written informed consent was sought from all participants before the start of all interviews.

### Study design

The discrete choice experiment was one component of a study of ANC staff knowledge, perceptions and attitudes towards treatment of malaria in pregnancy, and of pregnant women's acceptability of IST. The study setting and characteristics of the health facilities that participated in the trial have been described elsewhere [15]. The sampling scheme was designed to be regionally representative of ANC providers with 7 of 21 districts in the region randomly selected using probability proportional to size. The two districts involved in the Ashanti IST trial were specifically excluded from the sampling frame, therefore none of the health workers interviewed had been involved in the trial. All public and mission health facilities providing ANC services within these 7 districts were selected for the study, and all ANC providers present in these facilities on the day of the visit were invited to take part in the DCE. ANC staff were typically midwives and lower level cadres, including community health nurses and nursing/ward assistants.

### Designing the DCE questionnaire

In the DCE, respondents were repeatedly asked to choose between two alternative policy options each comprising a bundle of attributes representing SP-IPT and IST within an operational context. The choice of attributes included in the DCE was the result of qualitative work and discussions with key stake-holders [4], [16] concerned with the control of malaria in pregnancy (MiP) in Ashanti Region. A total of six attributes, each with two levels, were selected (Table 1).

**Table 1:** Description of attributes and levels, coding and priors on preferences.

ATTRIBUTE	LEVELS	Regression coding	Expected direction of coefficient
The type of approach to managing malaria in pregnancy	• preventive• curative (test and treat if parasite-positive)	• 0• 1	No prior
The anti-malarial drugs you have to prescribe to pregnant women	• SP(Fansidar)• Artesunate-amodiaquine (AS-AQ)	• 0• 1	Negative (preference for SP over AS-AQ)
Prevalence of anaemia for mothers treated with protocol	• 1 out of 100 women• 15 out of 100 women	• 1• 15	Negative (preference for better maternal outcomes)
Prevalence of low birth weight amongst infants of mothers treated with the protocol	• 10 out of 100 babies• 15 out of 100 babies	• 10• 15	Negative (preference for better health outcomes)
Staffing level for the ANC clinic	• Under-staffed• Adequately staffed	• 0• 1	Negative (preference for better staffing conditions)
The salary supplement included in the protocol	• GH. C10• GH. C20	• 10• 20	Positive (preference for higher bonus)

The first two attributes described the main components of the guidelines that staff may have to follow to manage malaria in pregnancy during ANC clinics: the type of approach and the drugs used. ANC staff could either adopt a preventative approach involving administration of a preventive drug to all women, or they could adopt a curative approach, involving the systematic screening of pregnant women with a rapid diagnostic test (RDT) and treating only those who were positive. The drug alternatives that they could give to pregnant women were artesunate plus amodiaquine (AS-AQ) given for three days or a single dose or SP. In addition, two attributes on the expected health outcomes of the management of malaria in pregnancy were included which were severe anaemia and low birth weight, the maternal and neonatal outcomes of primary concern to ANC providers [15]. The lower levels chosen for these two attributes were derived from the results of the trial in the Ashanti region, while the upper levels were based upon the sample size calculations for the trial which assumed that the baseline prevalence of severe anaemia and low birth weight amongst the study population of pregnant women and their newborns would be at least 12% and 6% respectively [14]. Finally, to provide a complete picture of the working conditions that may influence successful implementation of a policy two further attributes were included, one reflecting the workload and another the possibility of the health worker receiving a monthly bonus for their contribution to management of malaria. This bonus, ranging from 10 to 20 Ghana Cedis (at the time of the study, approximately USD 10 to 20), was approximately equivalent to 1.7–3.4% of an average salary for a midwife and chosen based on another recent IPT study in Ghana involving salary supplements for additional services [17]. The relevance and understanding of the first version of the tool was assessed through piloting with 4 ANC staff; after post- pilot discussions, rewording of the descriptions of the attributes was undertaken.

With two policy alternatives each comprising six attributes of two levels, the full factorial design (2^7^) included 128 pairs or choice scenarios. Since this would have been unmanageable for respondents, a fractional factorial design (i.e. a sub-set of this total number of possible combinations) was produced with SAS [18] to give 16 choice sets. A Street and Burgess ‘foldover’ design [19], in which choice sets systematically differ in all levels was avoided, as this design is more easily influenced by strong preferences for particular attributes. More precisely, we suspected that ANC staff would have strong preferences for either maternal or neonatal outcomes, and would systematically refuse to trade these two attributes for others, thus violating the compensatory decision-making process that is assumed of DCE respondents.

**Figure 1:**
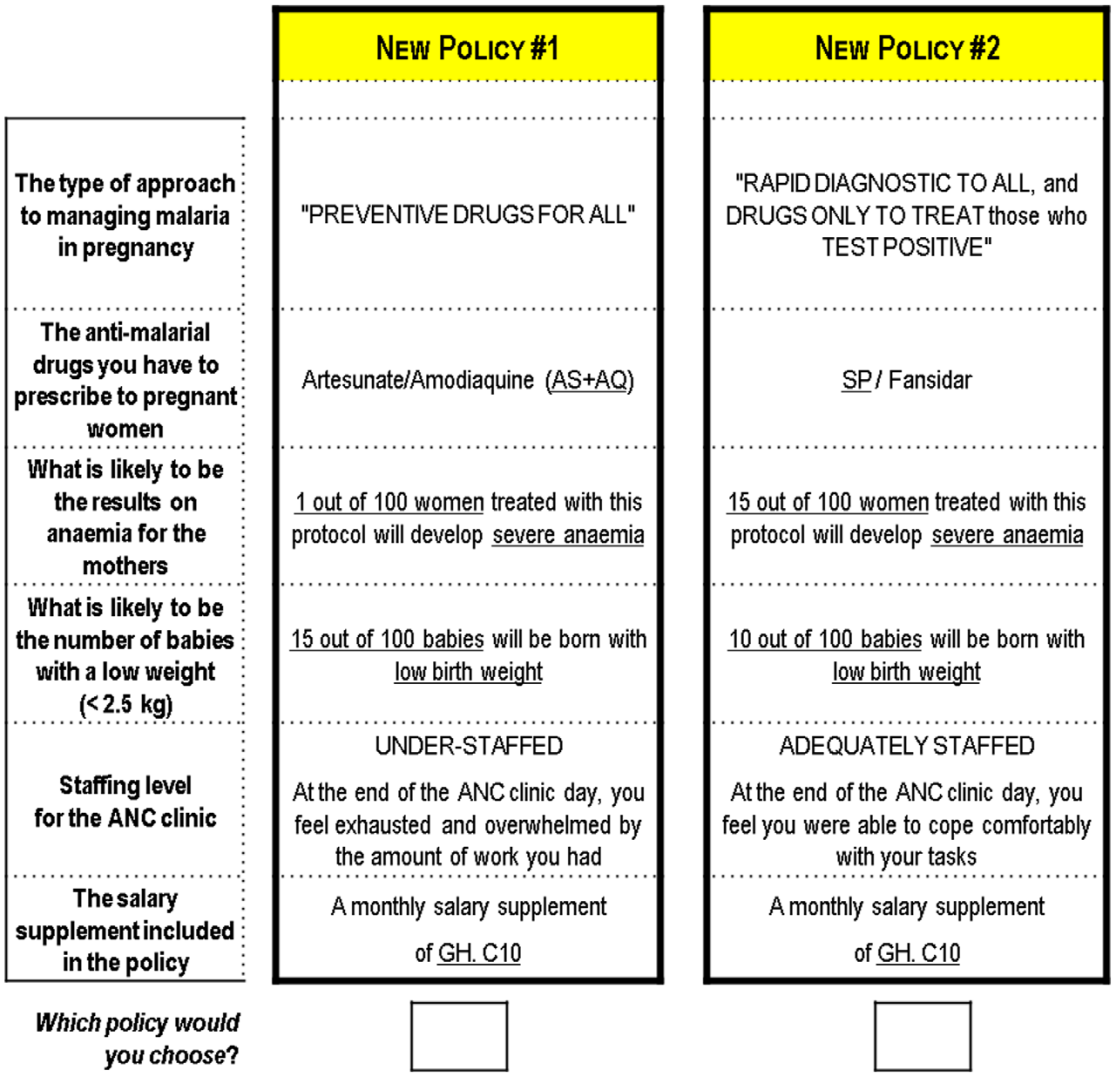
Example of a choice scenario.

Two sets of questionnaires were produced which differed in the order of the attributes, to test for potential order bias. An example of a choice scenario presented to participants is shown in Figure 1.

Enumerators were trained extensively to administer the questionnaire appropriately. To limit the expected reluctance or difficulty of ANC staff to conceptualise an alternative approach to the one in which they were currently trained and implementing, the interviewer mentioned the existence of on-going scientific studies whose results were likely to alter future guidelines of MiP control (in particular with regard to new drugs). To improve the salience of the task (i.e. make participants feel that they have some significant stake in the answers they give) and to reduce the hypothetical bias involved in lack of ‘consequentialism’ [20], respondents were told that the results of the task would be useful for informing future guidelines.

### Analysis

DCE analysis is rooted in random utility theory which assumes that the utility function of each health care worker is comprised of bundles of characteristics (denoted X) that define the MiP policy, as well as the individual's own preferences deriving from her personal characteristics (denoted Z). Therefore, when offered the possibility to choose between two alternative (and mutually exclusive) policies, *1* and *2*, a health care worker *n* will choose policy *1* if it is the one from which she derives the higher utility:
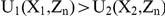
Using random utility theory, the indirect utility of each policy is given by: 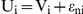 where V, the deterministic component of utility, is a function of the policy characteristics X_i_ and the individual characteristics Z_n_, and ε is the random component of utility accounting for the analyst's inability to accurately observe individual's behaviour [21].

Let y_n_* be a latent variable representing the difference in utility between the policies being compared for the respondent *n*. The dependent variable y_n_ is binary, reflecting whether respondent *n* chose policy 1 (variable coded 1) or policy 2 (variable coded 0). Therefore y_n_ = 1 if the latent variable y_n_*>0 with y* defined as a difference between the two policies:
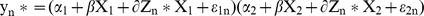
Taking differences for each pairwise choice (*k*), the equation to be estimated becomes:
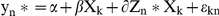
This regression model was estimated with NLOGIT 4.0 using a random effects logit model to account for the possible serial correlation in the 16 choices made by each respondent. When attributes were categorical variables (for example type of drugs or type of treatment approach used), dummy variables were used (Table 1).

Variability in preferences for different policy attributes according to respondents' demographic and training characteristics was tested by including interaction terms between policy characteristics and relevant individual characteristics in the regression model. For example an interaction between a categorical variable for age (being older than 40 years old) and the drug variable (which takes the value 1 for SP) measures the extent to which the preference for SP depends on age. When the inclusion of a series of interaction terms with a particular individual characteristic did not improve the overall goodness-of-fit of the model, the characteristic was not included in the final model.

Based on the coefficient estimates obtained in the model, the probability that respondents would choose a new MiP policy over the current one was simulated by computing the utility derived from each policy and the probability associated with that utility as follows:
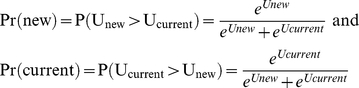
In a first series of simulations, the current guidelines in Ghana which recommend the SP-IPT approach are utilised, and according to the SP-IPT versus IST trial in the Ashanti Region, this scenario is associated with an approximately 1% prevalence of severe anaemia and a 10% prevalence of low birth weight [14]. Against this policy, it was assumed that new guidelines could be implemented that would reflect the current developments in clinical approaches to managing malaria in pregnancy [14], consisting of the introduction a new drug (AS-AQ) and possibly a different approach to control of MiP (curative as opposed to preventative). In a second series of simulations, the preferences of health workers were computed under a scenario of increased resistance to SP at a level that compromised the efficacy of the current policy, resulting in worse outcomes for mothers and their babies.

## Results

In the final model, all coefficients were in the expected direction (see priors in Table 1), with the exception of workload but this was not statistically significant (Table 2). Overall, ANC staff expressed a clear preference for a curative approach in which pregnant women would be tested and only treated if found positive, compared to the current preventive approach (OR 1.6; p = 0.001). However, there was some heterogeneity with regards to management approaches. Midwives and staff aged less than 40 years showed less preference for a diagnosis and treatment approach than for a preventive approach compared to lower level cadres including community health nurses and nursing/ward assistants (OR 0.64; p = 0.013) and older staff (OR 0.55; p = 0.002) respectively. Staff who had been working in the ANC department for a long time had less preference for a curative approach compared to staff who had spent less time in the ANC department (OR 0.65; p = 0.01). By contrast, there was no significant difference in preference for SP or AS-AQ (OR 0.90; p = 0.45), suggesting that the choice of drug to be used was irrelevant to ANC health workers as a whole; however, midwives were found to have a borderline significant preference for SP over AS-AQ compared to other cadres (OR 0.74; p = 0.07).

**Table 2:** ANC staff preferences for malaria management characteristics, and impact of age, professional status and experience on those preferences.

*VARIABLES*	*Coeff.*	*St. Err.*	*P-value*	*Odds-ratio*
Constant	0.055	0.055	0.318	1.056
Curative approach, IST [Preventive Approach, IPT]	0.487	0.151	0.001	1.628
Drug AS-AQ [SP]	−0.109	0.145	0.452	0.897
Under-staffing [Normal staffing conditions]	0.066	0.163	0.685	1.068
Low birth weight risk (per % point)	−0.201	0.017	0.000	0.818
Anaemia risk (per % point)	−0.131	0.007	0.000	0.877
Bonus (in GHC)	0.003	0.015	0.866	1.003
**Heterogeneity of preferences for a curative approach**				
Time in ANC (in years)	−0.434	0.169	0.010	0.648
Midwife [other ANC staff]	−0.442	0.178	0.013	0.643
Aged less than 40 y [aged 40 y or more]	−0.602	0.196	0.002	0.548
**Heterogeneity of preferences for AS-AQ**				
Time in ANC (in years)	−0.172	0.156	0.270	0.842
Midwife [other ANC staff]	−0.304	0.168	0.071	0.738
Aged less than 40 y [aged 40 y or more]	−0.062	0.183	0.737	0.940
**Heterogeneity of preferences for workload**				
Time in ANC (in years)	0.189	0.178	0.288	1.208
Midwife [other ANC staff]	0.217	0.189	0.251	1.242
Aged less than 40 y [aged 40 y or more]	0.073	0.206	0.723	1.076
**Heterogeneity of preferences for bonus**				
Time in ANC (in years)	0.028	0.017	0.105	1.028
Midwife [other ANC staff]	0.041	0.018	0.026	1.041
Aged less than 40 y [aged 40 y or more]	0.018	0.020	0.360	1.018
Log-likelihood	−1055.83			
Chi^2^	835.36***			
% correctly predicted	76.5%			

Note: ***p<0.001, **p<0.01, *p<0.05.

For categorical variables the reference category is indicated in brackets.

Interpretation: a negative coefficient indicates reduced preference for the level provided compared to the level in square brackets (for categorical variables) and a reduced preference for an increase in the variable (for quantitative variables).

The effect of intervention on low birth weight and severe anaemia appeared to be a very important attribute for ANC staff in choosing an intervention. Everything else being equal, increasing the incidence of low birth weight by 1 percentage point would reduce the odds of preferring that approach by 18%. A similar increase in maternal severe anaemia would translate into a decrease in the odds of preferring that approach by 10%. In terms of incentives, a larger bonus would increase the satisfaction of ANC health workers, particularly midwives. We found that preferences for various attributes of approaches to treating malaria in pregnant women did not vary depending on whether ANC staff had or had not attended specific training in the management of MiP. Consequently, we did not include this variable in the final model.

Preferences of the sampled health workers for the current policy situation (Policy 1) compared to the introduction of varying formats of new guidelines (Policy 2) were modelled. This modelling exercise showed that maintaining the current approach and changing SP for AS-AQ would be the preference of fewer health workers (46.2%) than changing to a curative approach whatever the drug of choice (SP 59.7%; AS-AQ 57.3%) (Table 3). These preferences would remain the same even with a higher workload. The introduction of a small bonus would have very little impact.

**Table 3:** Predicted preferences for changes to elements of the current guidelines that recommend SP-IPT for management of malaria in pregnancy.

Scenario	Policy 1	% preferring policy 1	Policy 2	% preferring policy 2
**1**	Current situation^a^	53.8	Policy 1 but different drug (AS-AQ)	46.2
**2**	Current situation^a^	40.3	Policy 1 but curative approach	59.7
**3**	Current situation^a^	42.7	Policy 1 but different drug (AS-AQ) and curative approach	57.3
**4**	Current situation^a^	41.3	Policy 1 but different drug (AS-AQ) and curative approach with higher workload	58.7
**5**	Current situation^a^	40.7	Policy 1 but different drug (AS-AQ) and curative approach with higher workload and small bonus (GHC10)	59.3

a Under the current situation, the guidelines for treatment are: preventive approach, use of SP, normal workload, no bonus. This has been shown to lead to the following health outcomes: 10% incidence of low birth weight and 1% incidence of severe anaemia.

In the second modelling exercise (Table 4), preferences of the health workers were estimated for the current situation (Policy 1) if resistance to SP increased leading to reduced efficacy of the current MiP guidelines and worse outcomes for babies and mothers. This policy was compared to the introduction of new clinical guidelines that would lead to good health outcomes for mothers and babies (Policy 2). The results show that ANC staff value the health outcomes of mothers and babies very highly over other attributes, as a vast majority would embrace new guidelines that would lead to significantly better health outcomes, irrespective of the drug, approach or workload (scenarios 6 to 9). A relatively greater resistance to change would be introduced if the improvements in health outcomes were perceived to be less (scenarios 10 and 11).

**Table 4:** Predicted preferences for changes to elements of the current guidelines, in the event that resistance to SP leads to worse health outcomes.

Scenario	Policy 1	% preferring policy 1	Policy 2: new guidelines (and good health outcomes^c^)	% preferring policy 2
**6**	Current guidelines with strong resistance to SP^a^	7.5	Policy 1 but different drug (AS-AQ) and good health outcomes	92.5
**7**	Current guidelines with strong resistance to SP^a^	4.8	Policy 1 but different drug (AS-AQ) and curative approach	95.2
**8**	Current guidelines with strong resistance to SP^b^	4.5	Policy 1 but different drug (AS-AQ) and curative approach with higher workload	95.5
**9**	Current guidelines with strong resistance to SP^a^	4.4	Policy 1 but different drug (AS-AQ) and curative approach with higher workload and small bonus (GHC10)	95.6
**10**	Current guidelines with mild resistance to SP^b^	29.3	Policy 1 but different drug (AS-AQ) and good health outcomes	70.7
**11**	Current guidelines with mild resistance to SP^b^	20.8	Policy 1 but different drug (AS-AQ) and curative approach	79.2

a Under this scenario, the clinical guidelines for treatment remain the same as currently defined, but resistance to SP would lead to worse health outcomes: 15% incidence of low birth weight and 15% incidence of severe anaemia.

b Under this scenario, the clinical guidelines for treatment remain the same as currently defined, but resistance to SP would lead to worse health outcomes: 13% incidence of low birth weight and 5% incidence of severe anaemia.

c Under all scenarios of policy 2, the health outcomes are 10% incidence of low birth weight and 1% incidence of severe anaemia.

**Figure 2:**
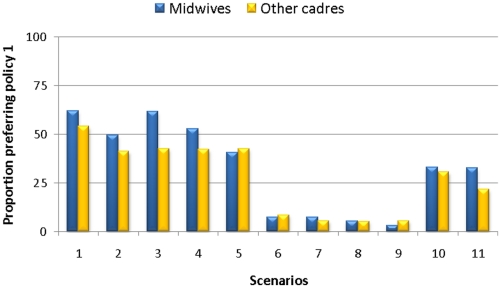
Comparison of potential resistance to changes in clinical guidelines from midwives and other ANC staff, under different changes to elements of the current guidelines.

As initial analyses showed that midwives had significantly different preferences to those of other cadres, a stratified analysis was undertaken (Table 5) on the proportion of midwives and other ANC staff who would prefer policy 1 across the simulations for the 11 scenarios (Figure 2). Midwives are more likely to stick to the current guidelines than lower level cadres (community health nurses and ward assistants) if health outcomes are equal between the two policy options (scenarios 1 to 5). However, if health outcomes are dramatically improved with new guidelines (scenarios 6 to 9), midwives would also overwhelmingly embrace them, and the simulations showed no differences between the two different cadres of health workers. However, midwives would still ‘resist’ (prefer current guidelines) more than the other cadres, if the improvements in health outcome were less impressive (scenarios 10 and 11).

**Table 5:** Midwives' and other cadres' preferences for malaria management characteristics, and impact of age and experience on those preferences.

	*MIDWIVES*				*OTHER CADRES*			
*VARIABLES*	*Coeff.*	*St. Err.*	*P-value*	*Odds-ratio*	*Coeff.*	*St. Err.*	*P-value*	*Odds-ratio*
Constant	−0.007	0.082	0.928	0.993	0.102	0.074	0.169	1.107
Curative approach, IST [Preventive Approach, IPT]	0.016	0.150	0.913	1.017	0.494	0.163	0.002	1.639
Drug AS-AQ [SP]	−0.498	0.138	0.000	0.608	−0.065	0.160	0.687	0.937
Under-staffing [Normal staffing conditions]	0.369	0.158	0.020	1.447	0.021	0.177	0.905	1.021
Low birth weight risk (per % point)	−0.218	0.025	0.000	0.804	−0.187	0.023	0.000	0.830
Anaemia risk (per % point)	−0.140	0.010	0.000	0.869	−0.124	0.009	0.000	0.883
Bonus (in GHC)	0.049	0.015	0.001	1.051	−0.002	0.017	0.899	0.998
**Heterogeneity of preferences for a curative approach**								
Time in ANC (in years)	−0.393	0.253	0.121	0.675	−0.476	0.228	0.037	0.621
Aged less than 40 y [aged 40 y or more]	−0.504	0.409	0.218	0.604	−0.601	0.222	0.007	0.548
**Heterogeneity of preferences for AS-AQ**								
Time in ANC (in years)	0.018	0.228	0.936	1.018	−0.349	0.215	0.104	0.705
Aged less than 40 y [aged 40 y or more]	−0.197	0.360	0.584	0.821	0.008	0.213	0.972	1.008
**Heterogeneity of preferences for workload**								
Time in ANC (in years)	−0.017	0.265	0.949	0.983	0.388	0.243	0.110	1.474
Aged less than 40 y [aged 40 y or more]	−0.016	0.415	0.969	0.984	0.049	0.237	0.837	1.050
**Heterogeneity of preferences for bonus**								
Time in ANC (in years)	0.020	0.026	0.448	1.020	0.037	0.024	0.123	1.037
Aged less than 40 y [aged 40 y or more]	0.001	0.040	0.987	1.001	0.022	0.023	0.338	1.022
Log-likelihood	−482.76				−569.46			
Chi^2^	453.37***				388.89***			
% correctly predicted	79.0%				75.5%			

Note: ***p<0.001, **p<0.01, *p<0.05.

For categorical variables the reference category is indicated in brackets.

Interpretation: a negative coefficient indicates reduced preference for the level provided compared to the level in square brackets (for categorical variables) and a reduced preference for an increase in the variable (for quantitative variables).

## Discussion

The DCE highlighted that health outcomes for mothers and babies were consistently more important to ANC staff than other attributes or elements of the alternative interventions for control of malaria in pregnancy in the Ashanti region of Ghana. This hierarchy of preferences means that introduction of a new intervention with clearly demonstrated health benefits for babies and mothers would result in very little resistance from staff. The study also revealed that ANC staff generally had a positive attitude towards a change in the approach to control of MiP from preventative treatment to parasitological diagnosis and treatment of malaria in pregnancy. There was no evidence of a difference in preferences between using SP or AS-AQ. However, there was heterogeneity in these preferences by age, cadre and experience. In particular, midwives were more resistant to potential changes to current guidelines than other groups. Respondents in the DCE were also surprisingly indifferent to a greater workload. This may be due to difficulties in conveying the notion of higher workload in a few words. However, in comparison to other attributes (in particular health outcomes for babies and mothers), workload was not a significant factor affecting staff preferences.

The overall implication for MiP control was that IST was seen as of equal preference to SP-IPT, likewise preference for SP was not significantly higher than for AS-AQ. Should the current policy be changed from SP-IPT to IST (for example, if SP resistance continues to increase and SP-IPT is no longer as effective), then improved health outcomes for mothers and babies is a strong factor that should be used in promoting effective provider uptake of IST.

Training was found to be a significant determinant of provider knowledge on SP-IPT and management of MIP in the health worker survey [15]; yet knowing the content of the guidelines does not necessarily translate into a strong preference for them, nor greater resistance to change. By contrast, professional status (whether one is a midwife or not) was associated with potentially greater resistance to change. The reluctance of midwives to use AS-AQ for treating malaria in pregnancy may be due to the fact that they are more aware of, or sensitive to, some of the side-effects associated with taking AS-AQ in pregnancy and see it as a stronger drug that can be justified for treatment of malaria but perhaps not for prevention [15]. Overall, we found that there is potentially a greater reservoir of resistance to change amongst midwives compared to lower levels of cadres. Less than a question of training, this may be due to the fact that midwives see themselves as the “repositories” of the views of the health authorities in ANC wards.

One of the limitations of the study design was the inability to describe workload in a detailed and realistic manner. In particular, because the DCE relied on a simple generic design, it was not possible to convey precisely the idea that IST, unlike IPT, could entail more work if staff were required to perform the RDT themselves. The levels of the workload attribute were necessarily paired with the two different approaches (IST and IPT). Thus a generic definition of workload was used, reflecting the idea that staff could be overwhelmed by their work or, on the contrary, could cope well with their workload. Whilst it may not reflect accurately the new tasks that would be required by IST, this qualitative description subsumes the precise issue of the test and recognises that workload is driven by more systemic issues than the obligation of undertaking particular tasks.

Although the use of hypothetical choices should be interpreted with caution, methodologically the hypothetical nature of the exercise has at least two advantages. First, the researcher has good control over the experimental design of the questionnaire, which ensured statistical robustness of the results and avoided confounding of preferences with other aspects of the MiP management approaches under investigation. Second, by allowing the inclusion of attributes that do not exist yet, DCEs can help researchers and policy-makers anticipate the effects of introducing new guidelines, and help them prepare to facilitate changes.

Discrete choice experiments have generally been shown to be reliable and valid, although this does depend on the context. Difficulties can arise if respondents do not understand the questions or the hypothetical situation in which they are asked to place themselves. In a follow-up question to the current choice experiment, 21% of respondents declared that they had experienced difficulties in making their choices. As suggested by feedback obtained during the pilot study, this might be linked to the unusual situation in which it placed respondents. While they are usually told what guidelines to implement, in the DCE they were asked to give their opinion about two different possible policies with different guidelines and outcomes. This may have led to a greater willingness to give “the right answer”. Yet, the defining of the “right answer” by the respondents in itself provides valuable information on what health care personnel think matters (e.g. is a better maternal outcome more important than the use of the “right” drug?). Other studies have suggested that respondents may not trade-off attributes but use more simple decision heuristics, in particular when some attributes are particularly meaningful and dominant compared to others. This could be the case here, considering the overwhelming importance given to health outcomes. However, given the *a priori* hypotheses, the results are plausible and support the theoretical validity of the technique. In addition, they largely concur with the findings of qualitative work undertaken with a similar population prior to this survey [15].

This study used an innovative application of an economic method to investigate the preferences of ANC staff for interventions in a context where testing IST would have required denying the national guideline of SP-IPT to pregnant women. The findings of the study provide insights into the attributes of these alternative interventions upon which ANC staff preferences are based. Health provider adherence to policy guidelines is a critical step in the translation of intervention efficacy to effectiveness. Identifying factors that will impede or promote adherence to new guidelines has the potential to improve the efficiency and effectiveness of training programmes for health workers and to reduce the gap between lives saved under trial conditions and those saved in an operational context.

This study was conducted when the preliminary findings of the trial showed that IST with either SP or AS-AQ was efficacious. The results of the DCE suggest that if the current guidelines were to be modified in Ashanti Region, resistance to change by antenatal clinic workers from a policy of SP-IPT to IST would be generally low. The relatively higher level of resistance shown by midwives amongst this group of health workers would be removed if health outcomes for the mother and baby were improved by the new strategy, as would be the case with increasing resistance to SP-IPT.

These results lead to the difficulty of convincing health workers that a new intervention is efficacious and should replace a current intervention. Encouraging evidence-based practice is complex [22], and there is a broad literature showing that the passive dissemination of information is generally ineffective [23]. Instead, proactive and interactive approaches tend to be encouraged. Although there is little evidence of effectiveness of these strategies in low-income settings [24], [25], a recent survey suggests that health care providers would be more likely to change clinical practice if research was conducted in their own country [26].

DCEs are a promising method for allowing the early identification of factors that will increase the likelihood of effective implementation of new interventions. Due to the fact that they rely on hypothetical scenarios, they do not replace operational research. But since they can be implemented whilst efficacy trials are still being conducted, and before policy guidelines are changed, DCEs can guide the focus and relevance of operational research by identifying at an early stage some potential obstacles to the effective implementation of new interventions. In the present application, we demonstrated that DCEs offer the potential to assess the preferences of providers for new MiP control guidelines including therapeutic regimens and patient management, as an alternative intervention to SP-IPT. We believe that DCEs have potentially wide applicability in improving the effectiveness of implementation of new interventions, in particular through the early identification of obstacles to the uptake of new interventions due to staff resistance. As such, DCEs can guide the design of subsequent implementation research or help advocate for support strategies that have the potential to mitigate staff resistance to a new policy.
